# Country of origin effects in explaining motivations for COVID-19 vaccine acceptance: A cognitive-affective-norm approach

**DOI:** 10.1016/j.heliyon.2024.e25901

**Published:** 2024-02-11

**Authors:** Svein Ottar Olsen, Ho Huy Tuu

**Affiliations:** a School of Business and Economics, UiT the Arctic University of Norway, N-9037 Tromsø, Norway; b Economics Faculty, Nha Trang University, 02 Nguyen Dinh Chieu, Nha Trang, Viet Nam

**Keywords:** Vaccine acceptance, Country of origin, COVID-19, Attitude theory, Vietnam

## Abstract

This study aims to broaden the understanding of the motivational factors influencing vaccine acceptance framed as product country image (PCI) by exploring the role of overall country image (OCI), vaccine knowledge and information inconsistency, in addition to the three most commonly investigated constructs: vaccine efficacy, fear of vaccine and social norm. A self-administered questionnaire was distributed online to 593 Vietnamese participants to collect data on COVID-19 vaccines from four different countries: the USA, the UK, Russia and China. Structural equation modelling was employed to test the proposed model and hypotheses. The results indicated that OCI had a positive effect on vaccine acceptance for vaccines from the USA and Russia, but a non-significant effect for vaccines from the UK and a negative effect for vaccines from China. Vaccine efficacy, social norm and subjective knowledge had a positive effect for most vaccines, while fear of the vaccine and information inconsistency had negative effects for certain vaccines. The results suggest that vaccine efficacy and social norm are more stable and significant predictors of vaccine acceptance than other constructs. Moreover, OCI moderated the effects of vaccine efficacy, fear of the vaccine, information inconsistency, subjective knowledge and social norm on vaccine acceptance for certain vaccines.

## 1 Introduction

Vaccination is considered a vital method of overcoming the COVID-19 pandemic [1]. Given the current availability of vaccines, Vietnamese citizens have the option of choosing from four different countries: AstraZeneca from the UK, Pfizer and Moderna from the USA, Sputnik from Russia and Vero Cell from China. This study contributes to the existing literature on vaccination acceptance [[2], [3], [4]] by distinguishing itself as the inaugural exploration that amalgamates the Cognitive-Affective-Norm framework [1] with the country of origin (COO) approach [5]. This unique theoretical approach explores the integration of vaccine efficacy, fear of the vaccine, social norms [1], knowledge [6,7], and information inconsistency [8,9] and how they collectively associate with COVID-19 vaccine acceptance across four distinct countries.

The product-country-image (PCI) reflects consumers' reflections, impressions, associations, stereotypes and evaluations of the quality of products made in a particular country [5]. COO is also defined as a general impression, overall country image (OCI) of a country's economy, culture, people, brands and products [5,10]. This study also contributes to the established literature [11,12] by delving into the potential moderating role of OCI in the relationships between motivational affective and cognitive beliefs (knowledge, information, efficacy, and fears) and social norms concerning vaccine acceptance. Notably, no prior research has examined the moderator role of OCI within the extended Cognitive-Affective-Norm framework [1], as far as our knowledge extends.

## 2 Theoretical framework

Several constructs have been used in the literature to express vaccine acceptance, including vaccine hesitancy, attitude, acceptance, intention, willingness, rejection, confidence, safety, demand, choice, or behaviour [6,7,13,14]. This study defines vaccine acceptance (or hesitancy) as a combination of individuals' attitudes towards a vaccine and their intention to take the vaccine. In this regard, the study follows traditional attitude theory [15] and measures attitude as an evaluation term of how positive or negative individuals are towards the vaccine, as well as their motivation (intention/willingness) to take the vaccine. Several studies have used either attitude [14,16], intention [13,[17], [18], [19]] or a combination of both [20] as indicators of vaccine acceptance.

Within the attitudinal framework, several theoretical approaches and factors are used to explore variations in individuals' attitudes and motivation to accept vaccines in general [6] and COVID-19 vaccines, in particular [21]. The Theory of Planned Behaviour (TPB) [7,15] is probably the most common theoretical framework. It is also recommended for future research to investigate the distinctions in the acceptance of various Covid-19 vaccines concerning the impact of conflicting information/media and Country-of-Origin (COO) on vaccine acceptance [1]. In existing literature, COO, product knowledge, and information have been amalgamated to explore their effects on product evaluation and purchase intention [22,23]. However, our understanding is limited regarding how COO can elucidate vaccine acceptance [2]. Furthermore, the influence of vaccine knowledge [6,7] and information [8,9,24] has been individually investigated. Consequently, our comprehension of the combined role they play in influencing vaccine acceptance is restricted. Moreover, previous studies primarily emphasize practical vaccine knowledge, with only a few incorporating subjective knowledge to explain vaccine acceptance [7]. Prior research also reveals contradictory effects of subjective knowledge and information on consumer acceptance [25]. Nevertheless, the manner in which psychological constructs like subjective knowledge and information inconsistency can impact vaccine acceptance remains unexplored.

**Fig. 1:**
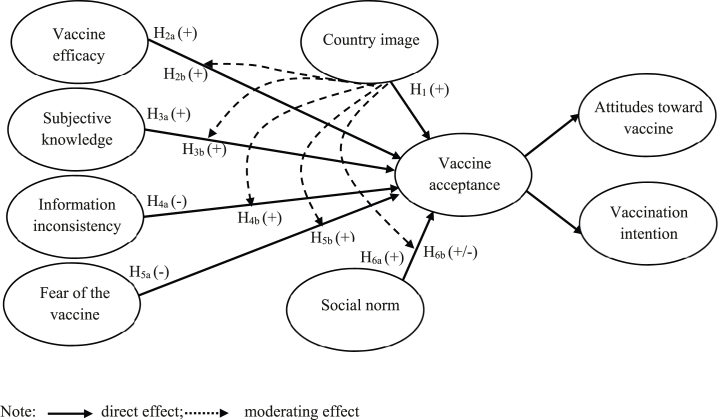
The proposed theoretical model.

Therefore, this study follows this traditional theoretical framework, focusing on whether and how cognitive (knowledge, information), affective (fear), efficacy and social norms are associated with COVID-19 vaccine acceptance. The theoretical model extends the previous Cognitive-Affective-Normative framework in the context of vaccine acceptance [1] by incorporating information inconsistency [8,9,26,27], subjective knowledge [6,7,26,28,29] and two facets of COO: product country image (PCI) and overall country image (OCI) [5]. The proposed model with hypotheses is presented in Fig. 1 and will be discussed below.

Thus, this study will extend the current literature by investigating if, how and why the COO effect influences vaccine acceptance. Do Vietnamese people have different acceptance of COVID-19 vaccines from different countries as a PCI-construct? Are their evaluations based on different cognitive (subjective knowledge and information inconsistency), affective (fear of the vaccine) or normative (social norms) motivational beliefs, and if so, how? What are the most important motivational factors in explaining variation in vaccine acceptance from different countries? Are PCI of the vaccine moderated by OCI – on all or on specific motivational beliefs?

### 2.1 Country of origin effects on vaccine acceptance

The consumer behaviour literature does not agree on how to define and measure country image or COO because the construct can be assessed on various dimensions, forms, level of abstractions and through multiple frameworks [5]. However, most studies seem to base their theoretical approach on attitude theory and measurement [30]. Our study contributes to the existing literature by combining two different but related constructs of country image in the context of vaccine acceptance. First, we define and measure product country image (PCI) as the acceptance (attitude and intention) of COVID-19 vaccines from four different countries (the USA, the UK, Russia and China) as the evaluation of a particular product or brand made in different countries [31, p. 404].

Secondly, the present study defines and measures overall country image (OCI) in accordance with its formal definition. OCI is an evaluation (overall impression, image, attitude, associations) of a country's economy, culture, people, brands and products [5,10]. Within an attitudinal theoretical framework [15], it can be the total of all cognitive or affective beliefs or associations one has about a particular country. Salient and relevant beliefs vary between individuals, brands, products, services, or other contextual issues. Our study frames OCI as evaluations associated with innovativeness, design, workmanship and prestige [31,32].

In the face of globalization, the emergence of hybrid products—those originating from two or more countries—adds an additional layer of complexity to framing or specifying country image constructs and its relationships to other constructs. In a recent meta-analysis of COO effects, De Nisco and Oduro [33] distinguish between OCI and more specific sub-components like country of manufacture, assembly, brand, and design. They found varying effects of these components on consumer behaviour, with the country of brand having the most significant impact on purchase intention. Their study also elucidates why the COO effect varies depending on whether the focus is on a specific product category (e.g., phones) or more general categories (e.g., electronics).

Consumer research shows various results about if and how OCI has positive, negative, or no effects on consumer expectations, attitudes, intention, behaviour, or loyalty toward products or brands (e.g., PCI) [34]. However, the general conclusions are that results show a positive effect of COO on consumer behaviour, but the results differ between consumers, countries, products and brands [30]. COO effect seems to be larger on perceived quality (attitudes) than on purchase intention and behaviour; differences in technological development, innovativeness, technological and economic performance are important for a positive COO effect [31].

The few studies that have investigated the role of COO in the context of (COVID-19) vaccine acceptance and hesitancy follow the main conclusion from the consumer behaviour and marketing literature [1,35,36]. Consumers are less willing to accept vaccines designed or manufactured by a country with a lower reputation than from a country with a high-quality reputation [18]. For example, Chiang et al. [36] found that Taiwanese are less likely to take a vaccine from China than from the US, Germany and Taiwan (home-country effect). Thus, this study suggests that:

H_1_: OCI is positively associated with vaccine acceptance (product country image).

The country-of-origin effect is suggested to have both a direct, mediation and moderating effect on health literacy and behavioural intentions [35]. COO can increase the attractiveness [37] and create strong emotional connections for products/brands from a country [38]. Thus, OCI can affect the consumer decision-making process and play a moderator role in the relationship between consumers’ specific beliefs or motivational forces and their acceptance, intention and consumption behaviour [11,12,32]. Therefore, this study contributes by exploring the moderator role of OCI in the motivational process of vaccine acceptance based on the Cognitive-Affective-Normative framework.

### 2.2 The cognitive-affective-norm framework and vaccine acceptance

As previously noted, our theoretical model extends the Cognitive-Affective-Normative framework in the context of vaccine acceptance by incorporating the cognitive component with information inconsistency and subjective knowledge in addition to perceived vaccine efficacy, fear of vaccines and social norms, as proposed by Pelegrín-Bornodo et al. [1].

#### 2.2.1 Perceived vaccine efficacy

Our study defines perceived vaccine efficacy as the belief that a vaccine will reduce the likelihood of infection or disease that can occur without the vaccine [1]. Both objective and subjective vaccine efficacy are crucial determinants of vaccine acceptance [6,8], including COVID-19 vaccines [1,8]. Previous studies also indicate that the effect of vaccine efficacy on vaccine acceptance varies (negative, positive, or non-significant) depending on the perceived levels of vaccine efficacy (low vs. high) [8]. Regardless of this evidence, low perceived vaccine efficacy may lead to low vaccine acceptance, while high perceived vaccine efficacy may lead to high vaccine acceptance. Thus, this study expects a positive effect of perceived vaccine efficacy on COVID-19 vaccine acceptance.

H_2a_: Vaccine efficacy has a positive effect on vaccine acceptance.

Furthermore, this study suggests that OCI can have a moderating effect on the relationship between vaccine efficacy and vaccine acceptance. Our suggestion is based on the rationale that a positive OCI can enhance consumer trust and reliability for products from a considered country, while a negative OCI can lower it [39,40]. In other words, the more positive the OCI is, the more confident consumers are about their vaccine evaluations, such as quality, safety and efficacy, leading to higher levels of positive attitudes and purchasing intention [39,40]. Thus, the next hypothesis is proposed:

H_2b_: OCI positively moderates the relationship between vaccine efficacy and vaccine acceptance.

#### 2.2.2 Subjective vaccine knowledge

Based on the literature on consumer knowledge [41], this study defines subjective knowledge about COVID-19 vaccines as an internal resource linking to several aspects, from perceived information stored in memory and the evaluation of vaccine quality, to what people know about the vaccines and how they can protect their health. The literature reveals that the relationship between subjective knowledge and vaccine acceptance is mixed (i.e., negative, positive, or nonsignificant) due to differences in knowledge measurements, research populations and contexts [7,26]. For example, previous studies show that parents who choose to vaccinate their children generally have limited knowledge and base their decisions on recommendations from others rather than on specific vaccine knowledge [6]. Some prior studies have demonstrated that attitude, risk, or side effects can mediate the relationship between knowledge and intention/choice of vaccines [29]. However, most studies find a positive association between individuals' knowledge of vaccines and their acceptance, hesitancy, intention, or uptake of (COVID-19) vaccines [17]. Thus, this study suggests:

H_3a_: Vaccine knowledge has a positive effect on vaccine acceptance.

The existing literature suggests that consumers often use OCI as an extrinsic cue for the country's product evaluations [32]; therefore, the formation of consumers' attitudes might be affected by OCI. During this evaluation process, attitude-strength attributes, such as product knowledge or involvement [40], are also formed and consolidated because OCI can help to guide, generate and amplify hierarchical levels of product/brand knowledge, such as product/brand awareness, familiarity, associations and reputation [32,42,43]. These discussions imply that OCI can moderate the relationship between product/brand knowledge and purchase intention. For instance, Maheswaran [44] reports that a novice is more likely to rely on OCI than an expert to form product evaluations, implying a negative interaction between OCI and product knowledge on outcomes such as attitudes, intention, or acceptance. Also, Hsu et al. [12] found that OCI enhances the positive effect of consumer attitudes on purchase intention. Although empirical evidence showing such a moderator effect of OCI on the effect of vaccine knowledge on vaccine acceptance is scant, this study suggests:

H_3b_: OCI positively moderates the relationship between vaccine knowledge and vaccine acceptance.

#### 2.2.3 Perceived information inconsistency

Perceived information inconsistency refers to an individual's subjective perception of discrepancies, contradictions, or conflicting information across various sources or channels of information related to a specific topic, event, or concept [45]. In the context of vaccine acceptance, perceived information inconsistency refers to the perception that the information available regarding vaccines, their effectiveness, safety, and other relevant factors, is contradictory or inconsistent across different sources such as media, health authorities, and public discourse [26,27]. This perception may lead individuals to feel uncertain, confused, or skeptical about the accuracy and reliability of the information, potentially influencing their decision-making process regarding vaccine acceptance.

The literature on the effect of information inconsistency on vaccine hesitancy is limited and ambiguous [27]. Inconsistent health information has been found to decrease acceptance and increase refusal for recommended prevention behaviours [45]. Moreover, concerns have been raised about the information inconsistency that has emerged during the COVID-19 pandemic and how it may negatively impact vaccine acceptance [26]. Perceived information inconsistency has also been found to increase vaccine concerns, scepticism and a lack of confidence about vaccines and vaccine risks, leading to vaccine hesitancy and refusal [14,26,27]. This negative effect can be exacerbated by the spread of vaccine-related misinformation, incomplete and inaccurate information, false beliefs and speculations through the internet and social media [46]. Building on these findings, this study proposes the following hypothesis:

H_4a_: Perceived information inconsistency has a negative effect on vaccine acceptance.

The literature also discusses the moderating effect of OCI in the consumer information process related to product evaluations and decisions [43,47]. In the product evaluation process, consumers can use different internal and external sources of product information [11]. When those sources are conflicting or inconsistent, especially when people are unfamiliar with the product, OCI may become an overall standard as a halo effect [10] to justify the accuracy of such information sources to affect consumer evaluations on specific product attributes [43]. This tendency is observed to be stronger when consumers' information processing ability in evaluating product attributes (e.g., benefits, risks, safety, or quality) is low or when their cognitive need for the product is high. Based on these discussions, the following hypothesis is suggested:

H_4b_: OCI negatively moderates the relationship between information inconsistency and vaccine acceptance.

#### 2.2.4 Fear of the vaccine

In this study, fear is defined as the degree to which individuals feel unsafe taking vaccines and fear the side effects [1]. Fear of the vaccine leads to negative cognitions and emotions that magnify vaccine risks and hinder vaccine benefits [45]. These perceived risks reduce confidence in new vaccines, increase concerns and contribute to vaccine hesitancy [48]. Fear of vaccine safety and adverse effects is among the primary reasons for vaccine rejection, including COVID-19 vaccines [49,50]. Recent studies have found that fear of the vaccine has a negative impact on COVID-19 vaccine acceptance [1,51]. Therefore, the following hypothesis is proposed:

H_5a_: Fear of the vaccine has a negative effect on vaccine acceptance.

Moreover, it can be argued that the relationship between fear of the vaccine and vaccine acceptance may be moderated by the OCI. OCI can influence consumer evaluations of vaccine safety and efficacy [34]. A positive/negative OCI can increase/decrease trust and confidence in the communication campaigns by government, healthcare systems, health institutions and authorities regarding the vaccine's safety and effectiveness [39,40]. Therefore, OCI can serve as a mitigating cue for people who are hesitant or fearful of vaccines and vaccination [52], thereby reducing the negative effects of fear of being vaccinated on vaccine acceptance [39,40]. As mentioned earlier, OCI has been found to enhance the positive effect of consumer attitudes on purchase intention [12]. Since fear of the vaccine is defined as a negative attitudinal construct, the moderating mechanism can be inferred in the opposite way. Thus, people are more likely to perceive vaccines from a country with a positive OCI as safe and effective, making their fears less consequential and, therefore, more likely to accept vaccination and vice versa [18]. Based on these discussions, the following hypothesis is suggested:

H_5b_: OCI negatively moderates the relationship between fear of the vaccine and vaccine acceptance.

#### 2.2.5 Social norm

Social norm in the context of vaccines refers to the social influence and pressure on people to either vaccinate or not with a particular vaccine [13]. If a person perceives that most people who want to take a vaccine also want them to take the vaccine, they might feel that it is a good idea to follow suit [53]. Conversely, if they learn that others are not willing to take the vaccine, they may doubt the idea of taking the vaccine [9]. Previous studies often indicate a positive effect of social norm on vaccine acceptance [54]. However, in a recent review, Limbu et al. [13] revealed that the effect of social norm on COVID-19 vaccine acceptance varies from low to high and can even be non-significant. The COVID-19 pandemic is still ongoing and most people (at least in Vietnam) believe that COVID-19 vaccines are an effective tool to overcome the pandemic. Because individuals are afraid of being infected, social norm could serve as a powerful social force to increase the acceptance of the vaccines [9]. Therefore, the next hypothesis is suggested:

H_6a_: Social norm has a positive effect on vaccine acceptance.

Previous studies also discuss country-of-origin effects as a complex construct based on the interactions of a wide range of cognitive, affective and normative motivations to affect the consumer buying decision process [55]. Overall, individuals tend to follow the social norm that is consistent with their perception of COO to make purchasing decisions [56]. For example, Hsu et al. [12] found empirical evidence showing that the positive relationship between subjective norm and intention is stronger for products with a more positive OCI in a retail context. It is also suggested that social norm may impact on consumer decisions to avoid products with a negative OCI [56]. In the vaccination context, it can be inferred that a positive OCI may amplify the effect of social norm while a negative OCI may reduce this effect related to vaccination. Since there is no evidence showing such a moderator role of OCI in the vaccine context, this study contributes by suggesting that:

H_6b_: A positive OCI has a positive moderator effect, while a negative OCI has a negative moderator effect on the influences of social norm on vaccine acceptance.

## 3 Methods

### 3.1 Research sample

This study employed an observational approach to assess the relationship between the intended constructs and individuals' acceptance of COVID-19 vaccination in a subset of the Vietnamese population. It followed the STROBE guidelines [57] to understand COVID-19 vaccination acceptance, especially in the context of achieving widespread immunity and controlling the pandemic.

This study utilized the snowball sampling technique and included respondents aged 18 years and older. However, individuals who had received two or more doses of COVID-19 vaccines were excluded from the study. The data collection began with initial participants who were known to the researchers. These initial participants, known as the “seeds,” were selected to represent a diverse range of characteristics related to the study. This choice helps ensure that subsequent referrals cover a broad spectrum of perspectives. To encourage honest responses and referrals, researchers committed to maintaining participant confidentiality, reducing concerns about sharing sensitive information. The process proceeded with initial participants referring other individuals who are relevant to the study's objectives. These referrals could include friends, family members, colleagues, or acquaintances who share similar characteristics or experiences. The process continued as each referred participant became a source of new referrals. This created a chain of participants who were interconnected through their relationships or networks [58]. The process continued until we reached a point of saturation. During this process, the data were analyzed daily until saturation was achieved, indicating that new participants were no longer providing significantly different results. At this stage, we had gathered a diverse range of viewpoints and experiences.

This study employed an online self-administered survey questionnaire to collect data from Vietnamese individuals via social networks such as Zalo and Facebook. As indicated by Baltar and Brunet [58], the number of cases detected through these social networks and the virtual response rate is higher than the traditional snowball technique because people increase their level of confidence due to their personal information (e.g., Facebook profiles) and also their participation in interest groups (e.g., Facebook groups). Moreover, the online questionnaire administration allows for quality control of the information and prevents case duplication. Therefore, the data collection strategy used in this study helped to reduce potential sources of non-response bias associated with using snowball sampling [58]. At the time of data collection (October 2021), the vaccination rate in the country had reached approximately 45% of people aged 18 and above. In the initial stages, only a small group of the highest priority population were vaccinated with Moderna or Pfizer, while the second highest priority groups were vaccinated with AstraZeneca. Later on, while the first two vaccines were still being distributed in limited amounts, Vero Cell occupied most of the market and Sputnik was in very limited supply. Therefore, regardless of people's expectations of receiving a certain vaccine, what they were vaccinated with was out of their control. Notably, in Vietnam, even though there is no compulsory stipulation for authors to declare whether the study obtained ethical approval, it is still advisable to include this information along with the rationale. Therefore, when conducting a study related to COVID-19 in Vietnam, obtaining ethical approval is a crucial step that ensures the research is conducted in a manner that upholds the well-being, rights, and dignity of the participants involved. Especially during the pandemic, various ethical considerations have arisen, making ethical approval essential for maintaining public trust, ensuring the credibility of research findings, and upholding the ethical principles that guide research involving human subjects. Since an online data collection mode was used, an indirect written consent must be obtained from the participants. Participants were instructed to read a consent form and check an agreement button to confirm their voluntary participation in the survey. Therefore, if they clicked on the disagreement button, the survey would come to an end.

To reduce biases in the estimated results due to common method variance, the survey items were ordered in a way that limited carryover and backfire effects [59]. Respondents were asked to rate all four different vaccines on one belief/item before moving on to the next. They were instructed to read a consent form and tick an agreement button to confirm their voluntary participation in the survey. The survey reached 787 participants, of which 649 (82.5%) participants agreed with the consent, and 138 (17.5%) disagreed with the consent. Among the participants who agreed with the consent, 56 were under 18 years old or had vaccinated two doses or more of the vaccines, leading them to the end of the survey. This study comprised all 593 participants who completed the entire survey. This sample size is considered very good (i.e., n > 500) [60] for an online survey and much larger than the maximum sample size of 250 compared to the used items [61], with 66.3% identifying as male, 33.7% being married, 66.6% being single, 63.1% residing in rural areas, 69.6% aged under 30, 93.3% completing 12 years or more of education, 63.4% practicing a religion, and 57.2% having an income below 11 million VND.

### 3.2 Construct measurements

*Vaccine acceptance* is operationalised to capture both vaccine attitude and vaccination intention. Vaccine attitude in this study is defined as a psychological tendency that is expressed by evaluating a particular vaccine in relation to a specific vaccine with some degree of favour/positive or disfavour/negative [15]. Accordingly, this study measured vaccine attitude using three items from prior studies [54]. Vaccination intention can be defined as the degree to which a person has formulated conscious plans to take a specific or not if and when that vaccine becomes available [15]. Most previous studies use one item such as “will get” [18], “likely to vaccinate” [16], or “willing to vaccinate” [19]. Thus, this study operationalised intention as a motivational construct and measured it using three items adapted from previous studies [16,18,19].

This study measured *vaccine efficacy* using three items, which were identified as major concerns of vaccination decision across the countries [62]. *Fear of the vaccine* (3 items) and *social* norm (3 items) using the scales previously validated by Pelegrín-Borondo et al. [1] for measuring these constructs in relation to a specific vaccine. Three items were used to measure perceived information inconstancy, reflecting inconsistent, contradictory and conflicting information about a COVID-19 vaccine [27]. Three items were used to measure *subjective knowledge*, one of which was sourced from the vaccine literature [7] and the other two were adapted from the consumer literature to capture perceptions of one's expertise about COVID-19 vaccines [63]. OCI was measured using four items capturing four basic features of OCI [32], reflecting a country's innovativeness, design, workmanship and prestige, all of which were used by previous studies [31].

A 7-point Likert-type scale ranging from “Totally disagree” to “Totally agree” was used for all scales. All measurements and items are shown in Table 1.

**Table 1:** Demographic characteristics.

	Frequency	%			Frequency	%
Gender				Marital status		
Male	393	66.3		Married	198	33.7
Female	200	33.7		Single	395	66.6
**Living place**				**Age**		
Rural	374	63.1		Under 30	413	69.6
Urban	219	36.9		From 30	183	30.4
**Education**				**Religion**		
<12 years	40	6.7		Yes	376	63.4
> = 12 years	556	93.3		No	217	36.6
**Income (VND)**						
Under 11 million	341	57.2				
From 11 million	255	42.8		**Total**	**593**	**100.0**

### 3.3 Analytical procedures

A confirmatory factor analysis was performed to evaluate the internal consistency, convergent validity and discriminant validity of the measurements [64]. Next, a structural equation model was used to estimate the proposed model. This study used Ping's [65] two-step estimation approach, developed for modelling interactions between latent variables, to test the hypotheses. The model fit was assessed using *χ*^*2*^ and three other fit indices: *RMSEA*, *GFI* and *CFI*. A good model fit was indicated by *RMSEA* <0.08 and *GFI* and *CFI* greater than 0.90 [66].

## 4 Results

### 4.1 Reliability and validity

The results (Table 2) indicate an acceptable fit of the measurement models for all four vaccines (*χ*^2^ = 627.16–666.78; *df* = 247, *p* = 0.000; *RMSEA* = 0.052–0.055; *GFI* = 0.911–0.923; *CFI* = 0.965–0.973). All of the factor loadings are bigger than 0.70 and significant (all p < 0.001). The composite reliabilities (all >0.80) and the extracted variances (all >0.60) are much larger than the suggested thresholds, indicating the reliability and validity of all constructs [61]. All correlations have an absolute value lower than 0.80 (maximum value = 0.787), which are smaller than the square root of average variance extracted from each pair of constructs AVEs (minimum value = 0.793) (Table 3), suggesting the discriminant validity of the constructs [67].

**Table 2:** Factor loadings, the reliability and extracted variance of the construct measurements.

Constructs/Items (*for vaccine X*)	Factor loadings				Composite reliability				Extracted variance			
	The USA	The UK	Russia	China	The USA	The UK	Russia	China	The USA	The UK	Russia	China
***Intention:****If vaccine X becomes available …*					0.95	0.95	0.96	0.94	0.87	0.87	0.88	0.83
I will take it	0.92	0.92	0.93	0.83								
I am likely to vaccinate it	0.94	0.95	0.94	0.93								
I am willing to vaccinate it	0.94	0.93	0.94	0.97								
***Attitudes:****I think …*					0.96	0.95	0.94	0.94	0.88	0.85	0.85	0.85
X is a good way to protect me from Covid-19	0.95	0.93	0.92	0.92								
X is safe	0.95	0.93	0.92	0.92								
X is highly effective	0.93	0.91	0.93	0.93								
***Vaccine efficacy***					0.95	0.94	0.94	0.96	0.87	0.85	0.86	0.88
With X, I will not being infected with Covid-19	0.96	0.95	0.96	0.96								
I am convinced of the efficacy of X	0.89	0.89	0.89	0.91								
I believe X will protect me from Covid-19	0.94	0.93	0.93	0.94								
***Knowledge:****Compared to an average person …*					0.91	0.91	0.91	0.92	0.77	0.77	0.78	0.79
I know a lot about the effectiveness and safety of X	0.90	0.90	0.89	0.91								
I know how to judge the quality of X	0.88	0.85	0.91	0.91								
I have a lot of knowledge about X	0.86	0.88	0.85	0.84								
***Information inconsistence:****Information about …*					0.90	0.84	0.84	0.83	0.76	0.64	0.63	0.63
… X is inconsistent	0.80	0.70	0.72	0.75								
… X is contradictory	0.90	0.83	0.81	0.75								
… X is conflicting	0.91	0.86	0.85	0.85								
***Fear of the vaccine***					0.88	0.83	0.89	0.89	0.71	0.63	0.74	0.75
I feel unsafe if I vaccinate X	0.78	0.69	0.79	0.81								
I am afraid of the temporary effects of X	0.87	0.85	0.90	0.89								
I am afraid of the permanent effects of X	0.87	0.83	0.88	0.89								
***Social norm***					0.98	0.98	0.98	0.98	0.79	0.80	0.79	0.77
People who are important to me think that I should use X	0.92	0.91	0.90	0.90								
People who influence me think that I should use X	0.88	0.89	0.86	0.88								
People whose opinions I value think that I should use X	0.88	0.89	0.90	0.86								
***Country image***					0.93	0.93	0.93	0.91	0.77	0.77	0.76	0.71
Country X's professional skills are creative	0.76	0.78	0.80	0.80								
Country X's designs are beautiful	0.89	0.92	0.91	0.87								
Country X is a prestigious country	0.92	0.92	0.91	0.85								
Country X has highly qualified workers	0.94	0.89	0.86	0.89								

*Notes.* All factor loadings are significant at *p* < 0.001 (all *t*-values >10.0).

**Table 3:** Correlations between constructs across vaccines.

Constructs	The USA’ vaccines							The UK's vaccines							Russia's vaccines							China's vaccines						
	1	2	3	4	5	6	7	1	2	3	4	5	6	7	1	2	3	4	5	6	7	1	2	3	4	5	6	7
1. Intention	–							–							–							–						
2. Attitudes	0.70	–						0.57	–						0.78	–						0.67	–					
3. Efficacy	0.57	0.75	–					0.43	0.75	–					0.67	0.78	–					0.60	0.77	–				
4. Knowledge	0.57	0.70	0.59	–				0.40	0.67	0.60	**-**				0.49	0.60	0.61	–				0.43	0.64	0.59	–			
5. Information	−0.45	−0.43	−0.37	−0.43	–			−0.60	−0.41	−0.34	−0.37	–			−0.60	−0.56	−0.52	−0.48	–			−0.57	−0.55	−0.50	−0.57	–		
6. Fear	−0.38	−0.20	−0.12	−0.12	0.21	–		−0.18	−0.14	−0.12	**−0.09**	−0.14	–		−0.46	−0.39	−0.39	−0.20	0.22	–		−0.53	−0.50	−0.57	−0.29	0.27	–	
7. Social norm	0.75	0.75	0.65	0.71	−0.43	−0.21	–	0.75	0.74	0.70	0.70	−0.42	**−0.08**	–	0.64	0.76	0.76	0.63	−0.57	−0.35	–	0.50	0.64	0.73	0.65	−0.57	−0.44	–
8. OCI	0.55	0.73	0.69	0.79	−0.12	−0.39	0.57	0.43	0.73	0.69	0.76	**−0.02**	−0.39	0.59	0.58	0.70	0.66	0.73	−0.47	−0.26	0.51	0.40	0.64	0.62	0.71	−0.36	−0.51	0.55
**Fit indices**																												
*Chi-squared (df)*	638.59 (247)							627.16 (247)							729.75 (247)							666.78 (247)						
*P*	0.000							0.000							0.000							0.000						
*GFI*	0.920							0.923							0.911							0.916						
*CFI*	0.973							0.971							0.965							0.972						
*RMSEA*	0.052							0.054							0.055							0.054						

*Notes.* Correlations in **bold** are nonsignificant at *p* < 0.05, and the remaining correlations are significant at *p* < 0.05; OCI: Overall country image.

### 4.2 Comparative means

The descriptive results with means and standard derivation of all constructs across four vaccines are presented in Table 4. Notably, there are consistency in the relative means of all constructs in the descending order, indicating the most positive evaluations for COVID-19 vaccines from the USA and the UK and the least positive evaluations for COVID-19 vaccines from Russia and China. For example, the OCI means of the USA, the UK, Russia and China are 6.19, 6.04, 5.69 and 4.95, respectively.

**Table 4:** Means and standardized deviations of the constructs across vaccines.

Constructs	The USA’ vaccines		The UK's vaccines		Russia's vaccines		China's vaccines	
	Mean	Std. deviation	Mean	Std. deviation	Mean	Std. deviation	Mean	Std. deviation
Intention	5.90	1.46	5.60	1.48	4.59	1.75	3.33	1.86
Attitudes	6.06	1.32	5.84	1.29	5.07	1.50	3.92	1.77
Vaccine efficacy	5.99	1.33	5.81	1.35	5.16	1.51	4.09	1.81
Subjective knowledge	5.41	1.58	5.32	1.52	4.66	1.64	4.36	1.81
Information inconsistence	3.08	1.78	3.25	1.64	3.73	1.59	3.85	1.64
Fear of the vaccine	2.73	1.71	2.83	1.68	3.16	1.72	3.85	1.89
Social norm	6.06	1.35	5.90	1.34	4.96	1.61	3.76	1.88
Overall country image	6.19	1.15	6.04	1.21	5.69	1.34	4.95	1.54

### 4.3 Testing hypotheses

The estimated results (Table 5) indicate that all four models have acceptable fit indices (*RMSEA* = 0.066–0.072; *GFI* = 0.825–0.841; *CFI* = 0.900–0.921).

**Table 5:** Testing proposed hypotheses and models across vaccines.

Structural effects and hypotheses					The USA’ vaccines		The UK's vaccines		Russia's vaccines		China's vaccines	
					Beta	*t-*value	Beta	*t-*value	Beta	*t-*value	Beta	*t-*value
***Direct effects***												
OCI (H_1_ +)					0.17	3.71***	0.06	1.41^ns^	0.11	2.53*	−0.09	−2.16*
Efficacy (H_2a_ +)					0.21	4.82**	0.46	7.31***	0.20	3.52**	0.46	7.10***
Subjective knowledge (H_3a_ +)					0.09	2.38*	0.00	0.11^ns^	0.18	4.39***	0.14	3.08**
Information inconsistence (H_4a_ -)					−0.05	−1.62^ns^	−0.08	−2.28*	−0.05	−1.29^ns^	−0.13	−2.86**
Fear of the vaccine (H_5a_ -)					−0.08	−2.24*	0.01	0.45^ns^	−0.03	−1.20^ns^	−0.07	−1.66+
Social norm (H_6a_ +)					0.50	9.93***	0.41	8.16***	0.43	7.13***	0.25	3.98***
***Moderating effects***												
OCI x Efficacy (H_2b_ +)					0.05	0.81^ns^	0.10	2.06*	0.06	1.01^ns^	0.21	4.57***
OCI x Knowledge (H_3b_ +)					0.04	0.46^ns^	−0.24	−5.06***	0.28	5.82***	0.18	4.44*
OCI x Information (H_4b_ +)					−0.05	−1.07^ns^	−0.02	−0.55^ns^	−0.12	−2.28*	0.10	2.53*
OCI x Fear (H_5b_ +)					0.12	3.69***	0.12	3.68***	0.02	0.84^ns^	0.14	3.89***
OCI x Social norm (H_6b_ +/−)					0.09	1.23^ns^	0.08	1.13^ns^	−0.12	−2.18*	−0.13	−2.71**
***Controlled effects***												
Gender					−0.03	−2.54*	0.04	0.61^ns^	0.00	0.14^ns^	0.00	0.01^ns^
Age					0.05	0.68^ns^	−0.06	−2.51*	−0.04	−0.73^ns^	0.01	−1.52^ns^
Education					0.00	0.82^ns^	0.03	1.81^ns^	0.08	1.93+	0.00	0.70^ns^
Income					0.03	1.73^ns^	0.02	1.35^ns^	0.00	0.12^ns^	0.02	−0.37^ns^
Married status					−0.06	−1.32^ns^	−0.04	−0.58^ns^	−0.05	−1.18^ns^	−0.06	−1.01^ns^
Living place					0.00	0.14^ns^	−0.03	−0.36^ns^	−0.02	−0.71^ns^	0.02	1.07^s^
Religion					−0.02	−0.93^ns^	0.00	−1.18^ns^	−0.03	−1.06^ns^	−0.03	−1.52^ns^
***High order construct of vaccine acceptance***												
Attitudes toward vaccine					0.94	18.47***	0.94	16.03***	0.94	17.60***	0.90	2018***
Vaccination intention					0.76	Fixed	0.72	Fixed	0.78	Fixed	0.78	Fixed
**Fit indices**												
*Chi-squared (df)*					2256.86 (578)		2370.86 (578)		2016.34 (578)		2055.07 (578)	
*P*					0.000		0.000		0.000		0.000	
*GFI*					0.911		0.900		0.919		0.921	
*CFI*					0.834		0.825		0.842		0.841	
*RMSEA*					0.070		0.072		0.066		0.066	
*R*^*2*^					85.7%		86.9%		77.1%		75.8%	

*Notes.*+*p* < 0.05 two tails; *p < 0.05; **p < 0.01; ***p < 0.001; ns: nonsignificant; OCI: Overall country image.

*Direct effects:* The present study investigated the effects of OCI, vaccine efficacy, fear of the vaccine, information inconsistency, subjective knowledge and social norm on vaccine acceptance for vaccines from the USA, the UK, Russia and China. The direct effects were analyzed and the results were partially in support of hypothesis H_1_, showing a positive effect of OCI on vaccine acceptance for vaccines from the USA (*β* = 0.17; *t* = 3.71; *p* < 0.001) and Russia (*β* = 0.11; *t* = 2.53; *p* < 0.05), but a non-significant effect for vaccines from the UK (*β* = 0.06; *t* = 1.41; *p* > 0.05) and a significant negative effect for vaccines from China (*β* = −0.09; *t* = −2.16; *p* < 0.05). Hypothesis H_2a_ was fully supported by a significant positive effect of vaccine efficacy across all vaccines (*β* = 0.20 ÷ 0.46; *t* = 3.52 ÷ 7.31; *p* < 0.001). The results also partially supported hypotheses H_3a_ and H_4a_, demonstrating a significant positive effect of subjective knowledge on vaccine acceptance for three cases (the USA: *β* = 0.09; *t* = 2.38; *p* < 0.05; Russia: *β* = 0.18; *t* = 4.39; *p* < 0.001; China: *β* = 0.14; *t* = 3.08; *p* < 0.01) and a significant negative effect of information inconsistency on vaccination acceptance for two cases (the UK: *β* = −0.08; *t* = −2.28; *p* < 0.05; China: *β* = −0.13; *t* = −2.86; *p* < 0.01). Furthermore, fear of the vaccine had a significant negative effect on vaccine acceptance for the USA (*β* = −0.08; *t* = −2.24; *p* < 0.05) and China (*β* = −0.07; *t* = −1.66; *p* < 0.05 two tails), partially supporting hypotheses H_5a_. Lastly, hypothesis H6a was fully supported by the data (*β* = 0.25 ÷ 0.50; *t* = 3.98 ÷ 9.93; *p* < 0.001), with a positive effect of social norm on vaccine acceptance across all vaccines.

*Moderating effects:* OCI was suggested to positively moderate the effects of vaccine efficacy (H_2b_), subjective knowledge (H_3b_), information inconsistency (H_4b_), fear of the vaccine (H_5b_) and social norms (H_6b_) on vaccine acceptance. The data partially supported all of these hypotheses. Specifically, the positive relationship between vaccine efficacy and vaccine acceptance was strengthened by a positive moderator effect of OCI for vaccines (H_2b_) from the UK (*β* = 0.10; *t* = 2.06, *p* < 0.05) and China (*β* = 0.21; *t* = 4.57, *p* < 0.001), but not for vaccines from the USA (*β* = 0.05; *t* = 0.81, *p* > 0.05) and Russia (*β* = 0.06; *t* = 1.01, *p* > 0.05). Additionally, the results revealed patterns for the positive effect of subjective knowledge on vaccine acceptance, with a positive moderator effect of CI (H_3b_) for vaccines from Russia (*β* = 0.28; *t* = 5.82, *p* < 0.001) and China (*β* = 0.18; *t* = 4.44, *p* < 0.001), but a negative moderator effect of OCI for vaccines from the UK (*β* = −0.24; *t* = −5.06, *p* < 0.001) and no effect for those from the USA (*β* = 0.04; *t* = 0.46, *p* > 0.05). OCI weakened the negative effect of information inconsistency on vaccine acceptance (H_4b_) for China's vaccines (*β* = 0.10; *t* = 2.53, *p* < 0.05), but amplified this negative effect for vaccines from Russia (*β* = −0.12; *t* = −2.28, *p* < 0.05), while having no impact on vaccines from the USA (*β* = −0.05; *t* = −1.07, *p* > 0.05) and the UK (*β* = −0.02; *t* = −0.55, *p* > 0.05). Furthermore, the negative effect of fear of being vaccinated on vaccine acceptance was reduced by a positive moderator effect of OCI (H_5b_) for vaccines from the USA (*β* = 0.12; *t* = 3.69, *p* < 0.001), the UK (*β* = 0.12; *t* = 3.68, *p* < 0.001) and China (*β* = 0.14; *t* = 3.89, *p* < 0.001), but not for those from Russia (*β* = 0.02; *t* = 0.84, *p* > 0.05). Lastly, OCI had a negative moderator effect on the positive relationship between social norms and vaccine acceptance (H_6b_) for those from Russia (*β* = −0.12; *t* = −2.18, *p* < 0.05) and China (*β* = −0.13; *t* = −2.71, *p* < 0.05), but not for vaccines from the USA (*β* = 0.09; *t* = 1.23, *p* > 0.05) and the UK (*β* = 0.08; *t* = 1.13, *p* > 0.05).

*Controlled variables’ effects:* Previous studies have shown that COVID-19 vaccine acceptance differs across countries and demographic characteristics such as income, age, gender, or education [68]. Therefore, this study includes these demographic characteristics as controlled variables (see Table 5). The results suggest that gender exhibited a negative effect (β = −0.03; t = −2.54) on vaccine acceptance only for vaccines from the USA, and age had a negative effect (β = −0.06; t = −2.51) on vaccine acceptance only for vaccines from the UK. Education had a positive influence on vaccine acceptance only for vaccines from Russia. Other characteristics (income, religion, marital status, and living place) had no effect on vaccine acceptance in all cases. These results are logical; for example, income does not make a difference as the vaccine is free in most countries. Regarding gender, there have been studies [69] that show that women can be more exposed to side effects than men. Notably, there were instances where controlled variables had no significant influence on vaccine acceptance in China.

## 5 Discussion

OCI is found to have a positive effect on vaccine acceptance for vaccines from the USA and Russia, a negative effect for vaccines from China and no effect for vaccines from the UK. These findings align with previous research that shows mixed relationships between OCI and attitudes, intention, or acceptance across products and services [34]. The results indicate that individuals tend to accept vaccines produced by countries with a positive OCI and are hesitant towards vaccines from countries with a less positive OCI, such as China. The mixed effects of OCI on vaccine acceptance may suggest that OCI's role in acceptance is not only direct but also mediated or moderated [37,38]. Therefore, this study's exploration of OCI's moderator effects provides a better understanding of how OCI can affect COVID-19 vaccine acceptance in the context of global vaccine competition [40].

The results show a positive effect of vaccine efficacy on vaccine acceptance across COVID-19 vaccines [1,8], implying that people value all vaccines as being effective and safe to accept. Notably, the relative importance of vaccine efficacy on vaccine acceptance is higher for vaccines from the UK (*β* = 0.46) and China (*β* = 0.46) than for those from the USA (*β* = 0.21) and Russia (*β* = 0.21). The result might come from the fact that vaccines from the USA (Moderna and Pfizer) were set as a priority for a small part of people working in public sections while vaccines from Russia were provided with a limited amount. Therefore, most people were vaccinated with vaccines from the UK and China, which increases the importance of vaccine efficacy on acceptance of those vaccines. These factors may also explain for the positive moderator effect of OCI on the effect of vaccine efficacy on acceptance for two vaccines from the UK and China but not for the USA and Russia. This suggests that contextual knowledge is sometime necessary to improve understanding of the country-of-origin effect in explaining vaccination acceptance [39,40].

The results indicate that subjective knowledge positively influences acceptance of vaccines from the USA, Russia and China but not from the UK. The findings are consistent with the current literature providing a positive but unstable relationship between subjective knowledge and vaccine acceptance [6,7,29]. The results accurately reflect the current state of COVID-19 vaccine provision and communication in Vietnam. It is worth noting that while the AstraZeneca vaccine from the UK was the first to be provided, many people received it without much prior knowledge about it. In contrast, other vaccines have been heavily promoted to convince people to accept them. Notably, under an interaction with OCI, the positive effect of subjective knowledge on vaccination acceptance becomes stronger for vaccines from Russia and China but becomes weaker for those from the UK and almost unchanged for those from the USA. The findings are important as the moderator role of OCI is almost underexplored by previous studies [12,44].

The positive moderator effects of OCI found for vaccines from Russia and China are consistent with the congruent perspective of the brand knowledge/equity [38] and the attitude strength-related moderators [32,40,43], reflecting the low levels of vaccination acceptance of both. In addition, the negative or nonsignificant moderator effect of OCI for vaccines from the UK or the USA may be affected by situational factors. The results seem to reveal that people might not necessarily consider both OCI and their knowledge to accept the vaccines from a country with the most positive OCI. They know or just favour all of the vaccines from the USA to make their vaccination decisions in a simple way. Also, vaccines from the USA (e.g., in spite of only a small amount) existed in Vietnam at the same time as those from the UK (AstraZeneca) and others had not been distributed. Thus, while most people wanted to be vaccinated with Moderna or Pfizer, they had to be vaccinated with AstraZeneca. This situation might lead to unexpected responses, causing a negative moderator effect of OCI for vaccines from the UK when compared to those from the USA (more positive OCI) and the UK (less positive OCI).

Information inconsistency is found to negatively affect vaccine acceptance from vaccines from the UK and China. Although previous studies often discuss the negative outcomes of vaccine information [14,26,27], empirical evidence is scant, highlighting our findings. Besides, it is worth noticing that the effect of information inconsistency is not significant for vaccines from the USA and Russia. The results are reasonable because most social networks in Vietnam tend to enhance the benefits but minimise the risks of vaccines from the USA, with almost no information about vaccines from Russia. While most people had to be vaccinated with those from the UK or China, conflicting information was automatically generated. Once again, the results reflect the status of the unequal distribution, leading vaccine hesitancy or refusal, especially for vaccines from China.

The study found that OCI had a positive moderating effect on vaccine acceptance for those from China, but a negative moderating effect on vaccine acceptance for vaccines from Russia. However, there was no moderating effect of OCI observed for the vaccines from the USA and UK on the relationship between information inconsistency and vaccine acceptance. The findings provide additional evidence to support the moderator role of OCI in information process to affect outcomes in the consumer behaviour literature [70,47]. Although this study expects a positive moderator effect, the valence of this moderator effect might depend on whether and how much OCI as extrinsic information could contribute to increase, decrease, or not contribute to inconsistency status [47]. For example, the findings may reveal that a negative OCI for Russia could enhance information inconsistency (others are positive) while a more negative OCI for China might decrease the consistency of information (others are negative).

Consistent with previous research [1,47], fear of the vaccine is found to negatively affect vaccine acceptance for vaccines from the USA and China. However, it has no impact on acceptance for vaccines from the UK and Russia, which is similar to the findings of some studies [51]. This result seems logical as AstraZeneca was the first vaccine to be provided popularly in Vietnam after a long time of living with the pandemic and Sputnik was almost absent from the distributed list of vaccines. Over time, some health risks occurred with most vaccines, leading to a fear of vaccines, negatively impacting on vaccine acceptance. However, the negative effect of fear of the vaccine was decreased by a positive moderator effect of OCI for most vaccines, as discussed by previous studies [18,52].

Social norm is found to strongly influence vaccine acceptance across the vaccines, which is similar to that of previous studies [13,54]. Compared with other antecedent constructs, social norm in this study was the most important determinant for COVID-19 vaccine acceptance. This result may reflect the fact that people were almost forced to take any available vaccine at the time of vaccination. Additionally, the findings showing the negative moderator effect of OCI on the social norm – vaccine acceptance relationship for vaccines from Russia and China – is important when such evidence is scant [12]. While social influences (e.g., such as advises and recommendations from doctors) are emphasised by previous vaccine studies as the main sources for vaccine acceptance [1], our findings further highlight the interactions between OCI and social norm on vaccine acceptance. Both social norm and OCI are extrinsic information and independent of cognitive and affective evaluations of vaccines (e.g., vaccine efficacy or subjective knowledge). The findings seem to reveal a conflict between these two sources of information, decreasing the effectiveness of social influences (e.g., health communication campaigns for encouraging to vaccinate a COVID-19 vaccine), especially for vaccines from a country with a negative OCI.

### 5.1 Practical implication

Vaccine efficacy is a crucial determinant of vaccine acceptance. Policymakers should therefore develop communication programs to convey the message of vaccine effectiveness, safety and health-protecting ability to increase acceptance rates. Public policy should limit information on health risks or deaths caused by unexpected vaccine effects, which can occasionally occur, in order to reduce people's fear of vaccines. This is vital because the safety and temporary and permanent effects of COVID-19 vaccines have not been fully established, despite recent developments. Notably, social norms are the most influential factor in vaccine acceptance. Therefore, communication strategies regarding vaccine efficacy and knowledge should be disseminated by key and influential figures, such as doctors, government sources, politicians, journalists, or others [1]. Moreover, inconsistencies in information about vaccines have been confirmed to decrease acceptance for certain vaccines, such as those from China. Although all vaccines appear to be effective and safe to some extent, policymakers should counter misinformation or disseminate corrective information about vaccines in the media environment to reduce noise [27], thus helping to increase vaccination acceptance, particularly for vaccines from countries with less positive OCI.

### 5.2 Limitation and future research

The present research is based on a non-representative Vietnamese consumer sample; therefore, future studies should use more representative samples. Therefore, caution should be exercised when extrapolating our findings to other parts of the Vietnamese consumer base as well as beyond the Vietnamese public. This study does not capture other antecedents (e.g., self-efficacy or perceived barriers) and potential moderators (e.g., involvement or ambivalence). Future studies can include those constructs to extend our knowledge on vaccine acceptance [13]. In addition, previous studies have shown that COVID-19 vaccine acceptance can be influenced by various factors, including prior vaccinations for viral infections like influenza [71]. Future research could benefit from the incorporation of additional constructs, such as individuals' history of influenza vaccination, preexisting comorbidities, previous COVID infection, reasons underlying the decision to either accept or decline the vaccines, perceptions of vaccine reliability, technology utilized, side effects associated with the manufacturing company, media coverage, personal preferences, and other factors [71]. Vaccine acceptance and vaccine hesitancy/refusal are opposite behaviours; thus, it would be advisable to replicate the research to explore whether the role of the intended construct on vaccine hesitancy/refusal differs. Both OCI and PCI can be assessed across various dimensions, forms, levels of abstraction, and as hybrid products [33]. Future research could, for example, evaluate the effects of Country of Design, Country of Manufacture, and Country of Brand as separate facets of PCI. Finally, this study uses self-reported behaviour and correlation methods on cross-sectional data, so the nature of the relationships is problematic. Experimental designs or longitudinal designs should be used to address issues of causality in future studies.

## 6 Conclusion

This study extends our understanding about the determinants of vaccination acceptance [2] by integrating both OCI and PCI [5] and extending previous studies to better capture the role of cognitive components in the CAN framework [1]. The findings confirm the most positive role of social norm, vaccine efficacy and to a certain extent, the positive role of subjective knowledge along with the negative effects of information inconsistency and fear of the vaccine in explaining vaccine acceptance. The findings also indicate that OCI has mixed moderator effects on these relationships for certain vaccines. The estimated models have relatively high explained variances (*R*^*2*^ = 77.1–86.9%), supporting the highly relevant combination of the motivational constructs.

## Ethics declarations

• This study was reviewed and approved by an independent committee of Nha Trang University, Vietnam, with the approval number: 21/QD-KKT-DHNT, May 15, 2022.
• All participants provided informed consent to participate in the study.
• All participants provided informed consent for the publication of their anonymised case details and images.

## Data availability statement

Data will be made available on request.

## Credit author statement

**Svein Ottar Olsen:** Writing – review & editing, Writing – original draft, Methodology, Conceptualization. **Ho Huy Tuu:** Writing – review & editing, Writing – original draft, Methodology, Investigation, Formal analysis, Data curation, Conceptualization.

Both authors have an equal contribution to this work.

## Declaration of competing interest

The authors declare that they have no known competing financial interests or personal relationships that could have appeared to influence the work reported in this paper.
